# MiR-146a-5p, targeting ErbB4, promotes 3T3-L1 preadipocyte differentiation through the ERK1/2/PPAR-γ signaling pathway

**DOI:** 10.1186/s12944-022-01662-6

**Published:** 2022-06-15

**Authors:** Yifen Wang, Jie Zhang, Xueru Chu, Mengke Wang, Yongning Xin, Shousheng Liu

**Affiliations:** 1 Department of Infectious Disease, Qingdao Municipal Hospital, Qingdao University, 1 Jiaozhou Road, Qingdao, 266011 Shandong Province China; 2 School of Medicine and Pharmacy, Ocean University of China, Qingdao, China; 3 Clinical Research Center, Qingdao Municipal Hospital, Qingdao University, 1 Jiaozhou Road, Qingdao, 266071 Shandong Province China

**Keywords:** 3T3-L1 cells, MiR-146a-5p, ErbB4, Preadipocyte differentiation

## Abstract

**Background:**

MicroRNAs (MiRNAs) are known to participate in preadipocyte differentiation, but the manner in which miR-146a-5p participates in this process remains unclear. This study was performed to examine the participation of miR-146a-5p in 3T3-L1 cell differentiation.

**Material and Methods:**

miR-146a-5p expression was upregulated and down-regulated to examine effects on 3T3-L1 cell differentiation. Bioinformatics analysis was performed to predict its target genes, and the signaling pathway it regulates was identified by qRT-PCR and Western blotting. The expression of miR-146a-5p in epididymal adipose tissue from obese mice and in an obese mouse adipose cell model was examined by qRT-PCR.

**Results:**

3T3-L1 cells differentiated into mature adipocytes successfully, as verified by increased areas of intracellular lipid droplets and elevated expression of mature adipocyte markers, and these cells had elevated miR-146a-5p expression. The intracellular lipid droplet and triglyceride contents and the expression of mature adipocyte markers were significantly increased in miR-146a-5p–overexpressing 3T3-L1 cells and markedly decreased in miR-146a-5p–inhibited 3T3-L1 cells. ErbB4 was a predicted target gene of miR-146a-5p. In miR-146a-5p–overexpressing 3T3-L1 cells, ErbB4 expression and ERK1/2 phosphorylation were decreased and the expression of PPAR-γ was increased; the opposite was observed in miR-146a-5p–inhibited 3T3-L1 cells. In addition, miR-146a-5p expression was significantly increased in the mouse epididymal adipose tissue and adipose cell model.

**Conclusions:**

Upregulated miR-146a-5p expression was related to 3T3-L1 cell differentiation. MiR-146a-5p promoted 3T3-L1 cell differentiation by targeting ErbB4 and via the ERK1/2/PPAR-γ signaling pathway.

**Supplementary information:**

The online version contains supplementary material available at 10.1186/s12944-022-01662-6.

## Introduction

Obesity, defined as BMI > 30 kg/m^2^ in clinical practice, is traditionally manifested as excessive body fat, which has adverse health effects [1]. It correlates strongly with the development of cardiovascular disease, type 2 diabetes, dyslipidemia, and other diseases [2]. A survey of a sample representing 77.5% of the global population revealed that obesity has become significantly more prevalent among boys and girls, increasing from < 1% in 1975 to 6–8% in 2016; it has also increased among women (from 6 to 15%) and men (from 3 to 11%) [3]. Obesity has become a serious social burden, and the risks of obesity-related diseases have increased in recent years. Thus, elucidation of its etiology is urgently needed to achieve effective clinical prevention and treatment.

The expansion of adipose tissue occurs mainly through preadipocyte differentiation (hyperplasia) and/or hypertrophy [4]. Both processes are regulated positively or negatively by many factors, including hormones, signal transduction pathways, delta-like non-canonical notch ligand 1–1 (an epidermal growth factor repeat-containing inhibitor), extracellular matrix components, and transcription factors [5]. The regulation of preadipocyte differentiation is generally considered to be a positive mechanism to prevent metabolic changes [6]. Mouse 3T3-L1 preadipocyte differentiation, used in the most common model of this process [7–9], is a complex physiological procedure resulting in increased fatty acid binding protein 4 (FABP-4), peroxisome proliferator-activated receptor γ (PPAR-γ), sterol regulatory element binding protein 1c (SREBP-1c), and adipogenic marker gene expression [10–12].

Recent research has shown that some miRNAs participate in adipocyte differentiation; miR-16-5p and Bat-miR-204 promote it, and miR-125a-5p and miR-23a inhibit it [13–16]. MiR-146a-5p was found to inhibit the differentiation of intramuscular preadipocytes via Akt/mTORC1 signaling in a porcine model [17], whereas Francisco et al. [18] observed elevated miR-146a-5p expression in differentiated human adipocytes. This miRNA is known to participate in the pathological processes of multiple metabolism-related diseases and obesity [19–24], but its role in adipocyte differentiation remains unclear.

Epidermal growth factor receptor-4 (EGFR4, also referred to as ErbB4), a type I transmembrane receptor tyrosine kinase, belongs to the Tyr protein kinase family and EGFR superfamily [25]. After binding to neuroregulatory proteins and other factors, this type of protein is activated and can induce various cellular responses, including mitosis and differentiation [26–28]. ErbB4 has been linked to the risks of type II diabetes and metabolic syndrome [29]. Zeng et al. [30] found that its deletion predisposed mice to metabolic syndrome, and observed reduced ErbB4 expression in differentiated 3T3-L1 cells. Many studies have shown that ErbB4 upregulates the phosphorylation of MAPK42/44 (also called ERK1/2) [31–34], a significant negative regulator of adipogenesis [35–37]. Bioinformatic analyses suggest that ErbB4 is a target gene of miR-146a [38].

This study was conducted to investigate the role of miR-146a-5p in adipocyte differentiation. This study found that miR-146a-5p expression increased significantly during 3T3-L1 differentiation. By upregulating and inhibiting the expression of miR-146a-5p, this study demonstrated that miR-146a-5p promotes 3T3-L1 cell differentiation. Molecular mechanism studies suggested that miR-146a-5p interacts with ErbB4 and promotes preadipocyte differentiation through the ERK1/2/PPAR-γ signaling pathway. Moreover, in vivo studies showed that upregulated miR-146a-5p expression correlated with adipogenesis in mice.

## Materials and methods

### Cell culture and differentiation

3T3-L1 cells (purchased from Shanghai FuHeng Biotechnology Company Limited, Shanghai, China) were cultured in Dulbecco’s modified eagle medium (DMEM; HyClone, Logan, UT, USA) with 10% newborn calf serum and 1% penicillin–streptomycin (P/S) supplementation in an incubator at 37 °C with 5% CO_2_. Cell differentiation was conducted using a modification of a previously reported protocol [39]. Briefly, the cells were inoculated in six-well plates at a density of 5 × 10^5^/well. When the density reached complete confluence, the cells were subjected to contact inhibition for 2 days, and were then considered to be at day 0 of induced differentiation. The cell medium was replaced with medium 1 [89% DMEM, 10% fetal calf serum (FBS), 1% P/S, 1 μM dexamethasone, 5 μg/mL insulin, 2 μM rosiglitazone, and 0.5 mM 3-isobutyl-1-methylxanthine] and cultured for 48 h. Then, the cell medium was replaced with medium 2 (89% DMEM, 10% FBS, 1% P/S, and 5 μg/mL insulin) and cultured for 48 h, followed by replacement with complete medium (89% DMEM, 10% FBS, and 1% P/S) every 2 days during culture for 8 days.

### Quantitative real-time polymerase chain reaction

RNAex Pro reagent (Accurate Biology, Changsha, China) was used to isolate total RNAs from the 3T3-L1 cells. MiRNAs were reverse transcribed to cDNAs using a commercially available kit (TIANGEN, Beijing, China), and mRNAs were reverse transcribed to cDNAs using the M-MLV kit (Accurate Biology). The relative concentration of miR-146a-5p was measured by quantitative real-time polymerase chain reaction (qRT-PCR) using a miRNA qPCR kit (TIANGEN) and normalized to U6. The expression of mature adipocyte marker genes was examined using a SYBR Green PCR kit (QIAGEN, Germany) and normalized to β-actin. The primer sequences are listed in Table 1.

**Table 1:** qRT-PCR primer sequences

Gene	Primer Sequences
Has-miR-146a-5p	F: CD201-0013 (TIANGEN, Beijing, China) R: R7015 (TIANGEN, Beijing, China)
U6	F: CD201-0145 (TIANGEN, Beijing, China) R: R7015 (TIANGEN, Beijing, China)
DLK1	F: 5’-GAAAGGACTGCCAGCACAAG-3’ R: 5’-CACAGAAGTTGCCTGAGAAGC-3’
ErbB4	F: 5’-GTGCTATGGACCCTACGTTAGT-3’ R: 5’-TCATTGAAGTTCATGCAGGCAA-3’
FABP-4	F: 5’-GATGAAATCACCGCAGACGACA-3’ R: 5’-ATTGTGGTCGACCTTTCCATCCC-3’
PPAR-γ	F: 5’-AAGAGCTGACCCAATGGTTG-3’ R: 5’-GCTTTATCCCCACAGACTCG-3’
SREBP-1c	F: 5’-GATCAAAGAGGAGCCAGTGC-3’ R: 5’-TAGATGGTGGCTGCTGAGTG-3’
*β*-Actin	F: 5’-CAGCTTCTTTGCAGCTCCTT-3’ R: 5’-CACGATGGAGGGAATACAG-3’

*Abbreviations*: *F* Forward, *R* Reverse, *DLK1* Delta-like non-canonical notch ligand 1, *ErbB4* Erb-b2 receptor tyrosine kinase 4, *FABP-4* Fatty acid binding protein 4, *PPAR-γ* Peroxisome proliferator activated receptor gamma, *SREBP-1c* Sterol regulatory element binding transcription factor 1

### Oil Red O staining

3T3-L1 cells were inoculated in six-well plates at a density of 4 × 10^5^/well. Oil Red O (ORO) staining was applied using a commercially available kit (Solarbio, Beijing, China). The ImageJ software was used to calculate the lipid droplet area, and the cell numbers in photographs of the samples were recorded. The average lipid accumulation in each cell was calculated as the overall area divided by the overall cell number. Thereafter, 300 μL 100% isopropyl alcohol was added to each well for 5 min to extract the lipids, the solution was diluted 20 times, and absorbance at 510 nm was read using a microplate reader.

### Western blotting analysis

Cells cultured in the six-well plates were collected into 1.5-mL tubes. The cells were washed two times with phosphate buffer solution and lysed with 100 μL radio immunoprecipitation assay lysis buffer (containing 1 μL cocktail and 1 μL phosphatase inhibitor) with vortex vibration for 5 min; then, the tubes were placed on ice for 30 min. The solutions were centrifuged at 12,000 × *g* and 4 °C for 30 min to collect the supernatant (cell total proteins). Then, 5 × sodium dodecyl sulfate (SDS) containing β-mercaptoethanol was added at a 4:1 ratio and the samples were placed in a 100 °C water bath for 5 min. They were then subjected to 12% SDS polyacrylamide gel electrophoresis and transferred to polyvinylidene fluoride membranes. Then the membranes were blocked with 5% bovine serum albumin (BSA) at 25 °C for 1 h and incubated with primary antibodies at 4 °C for 10 h. Anti–β-actin (1:3000; Boster, China), anti–SREBP-1 (1:500; Absin, Shanghai, China), anti–delta-like non-canonical notch ligand 1 (DLK1; 1:1000; Sangon Biotech, China), anti-ErbB4 (1:1000; Santa Cruz Biotechnology, USA), anti–FABP-4 (1:750; Beyotime, China), anti–PPAR-γ (1:1000; Proteintech, China), anti–p-ERK1/2 (1:2000; Proteintech), and anti-ERK1/2 (1:1000; Proteintech) were used. After incubation, the membranes were washed three times with Tris–HCl buffered saline containing 0.1% Tween -20 (TBST). Then, they were incubated with secondary antibodies (1:5000) at 25 °C for 1 h. After washing three times with TBST, the expression of each gene was detected by chemiluminescence (4800 Multi; Tanon, Shanghai, China) using ECL reagent (Millipore, USA).

### Identification of miR-146a-5p target genes

The prediction of potential miR-146a-5p target genes was performed using the miRDB (http://mirdb.org/), miRTarBase (http://mirtarbase.mbc.nctu.edu.tw/php/search.php), miRWalk (http://mirwalk.umm.uni-heidelberg.de/), and TargetScan (http://www.targetscan.org/vert_71/) online databases. The final intersection of genes was represented using VennDiagram. KEGG and GO enrichment analyses of the target genes were performed using the clusterProfiler program of the RStudio software, with screening for genes potentially involved in cell differentiation. ErbB4 was selected as the miR-146a-5p target gene in 3T3-L1 cells. The TargetScan database was used to predict binding sites of miR-146a-5p–ErbB4, and the regulatory role of miR-146a-5p for ErbB4 was verified by Western blotting and qRT-PCR analyses.

### Experimental animals

Eight-week-old male C57BL/6 mice were housed in a specific pathogen–free animal facility. They were divided into a control group fed a normal diet and an obesity group fed a high-fat diet (HFD; *n* = 6 each) for 16 weeks. The mice were sacrificed after fasting for 6 h. The epididymal fat of each mouse was rinsed and its weight was recorded, followed by fixation with 4% paraformaldehyde for hematoxylin–eosin (HE) staining of tissue sections. Adipocyte size was observed under a microscope, and the adipocyte area was measured using Image J software. Total RNAs were extracted from the epididymal fat and the relative concentration of miR-146a-5p was examined by qRT-PCR. All animal experiments were conducted in compliance with the guidelines of the Medical Laboratory Animal Care Committee of Qingdao University (Qingdao, China).

### Establishment of an obese mouse adipose cell model

The obese mouse adipose cell model was established as described by Yu et al. [40]. Specifically, differentiated 3T3-L1 cells were stimulated with 0.3 mM palmitic acid (model) or BSA (control). After 48 h, their morphology was observed by ORO staining, and the intracellular triglyceride (TG) content was measured using a TG assay kit (Nanjing Jianchen, China). qRT-PCR was performed to determine relative miR-146a-5p concentrations.

### Statistical analysis

All quantitative data are presented as means ± standard deviations (SDs) from three independent replicates. Differences between two groups were analyzed using Student’s *t* test. GraphPad Prism (version 9; GraphPad Software, CA, USA) was used for data analysis. *P* < 0.05 was considered to be significant.

## Results

### MiR-146a-5p upregulation correlated positively with 3T3-L1 cell differentiation

**Fig. 1:**
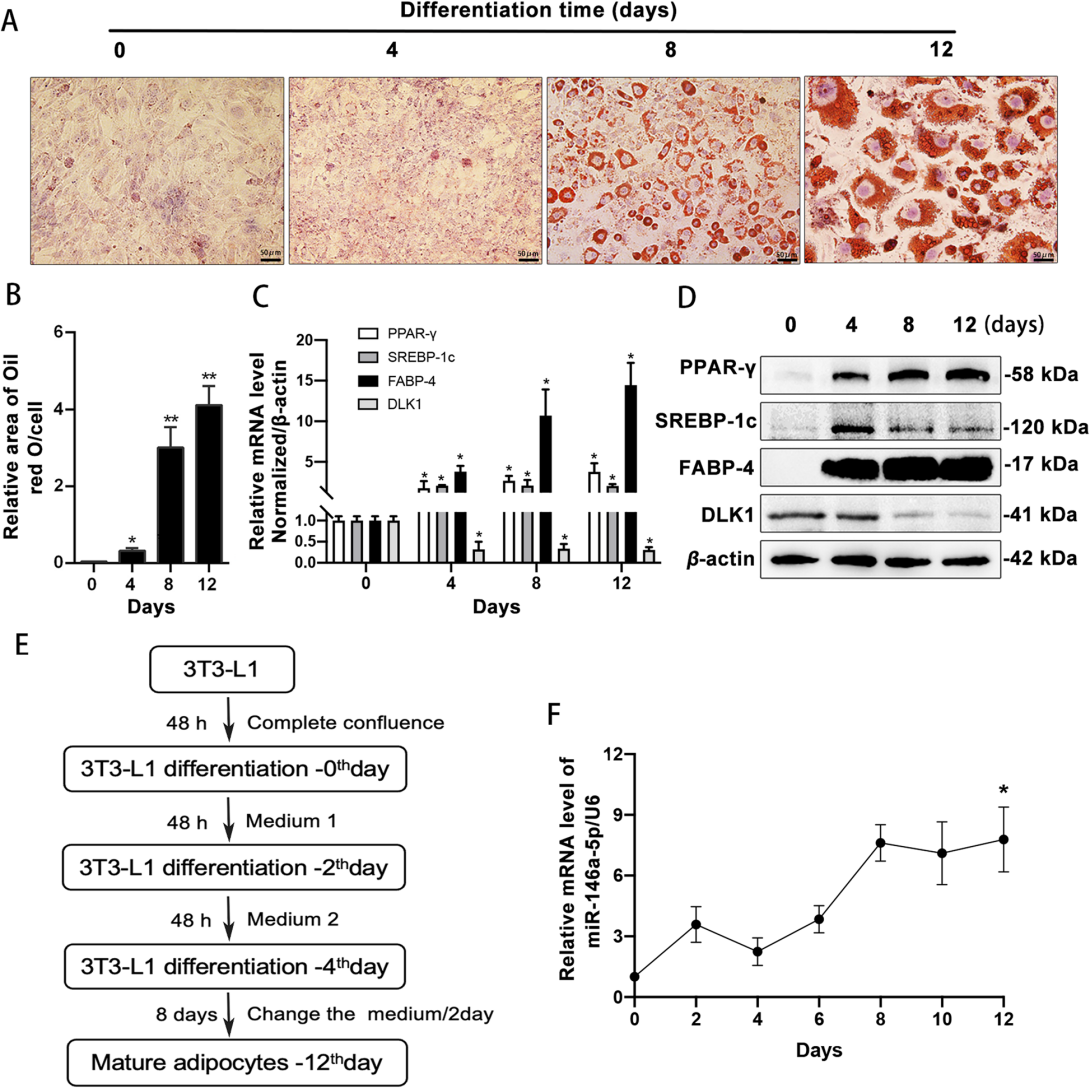
Differentiation of 3T3-L1 cells and expression of miR-146a-5p during the process. **A** Oil Red O staining of 3T3-L1 cells at different differentiation timepoints (200 ×). **B** Relative lipid droplet areas in 3T3-L1 cells at different differentiation timepoints. **C** Relative mRNA expression of adipogenesis-related genes during 3T3-L1 cell differentiation. *P < 0.05 vs. undifferentiated 3T3-L1 cells. **D** Protein levels of adipogenesis-related genes during 3T3-L1 cell differentiation. **E** Flowchart of 3T3-L1 cell differentiation. **F** Relative expression of miR-146a-5p during 3T3-L1 differentiation. Data in (**B**), (**C**), and (**F**) are means ± SDs (*n* = 3)

The 3T3-L1 cells enlarged gradually during differentiation into mature adipocytes, and ORO staining revealed markedly increased intracellular lipid droplet content (Fig. 1A and B). The expression of mature adipocyte markers (PPAR-γ and FABP-4) increased gradually and that of the pre-adipocyte marker DLK1 decreased gradually at the protein and mRNA levels. SREBP-1c expression also increased at these levels during differentiation (Fig. 1C and D). Thus, the 3T3-L1 cells differentiated into mature adipocytes successfully. In addition, miR-146a-5p expression increased significantly during 3T3-L1 differentiation, indicating positive correlation of these processes (Fig. 1E and F).

### MiR-146a-5p promoted 3T3-L1 cell differentiation

**Fig. 2:**
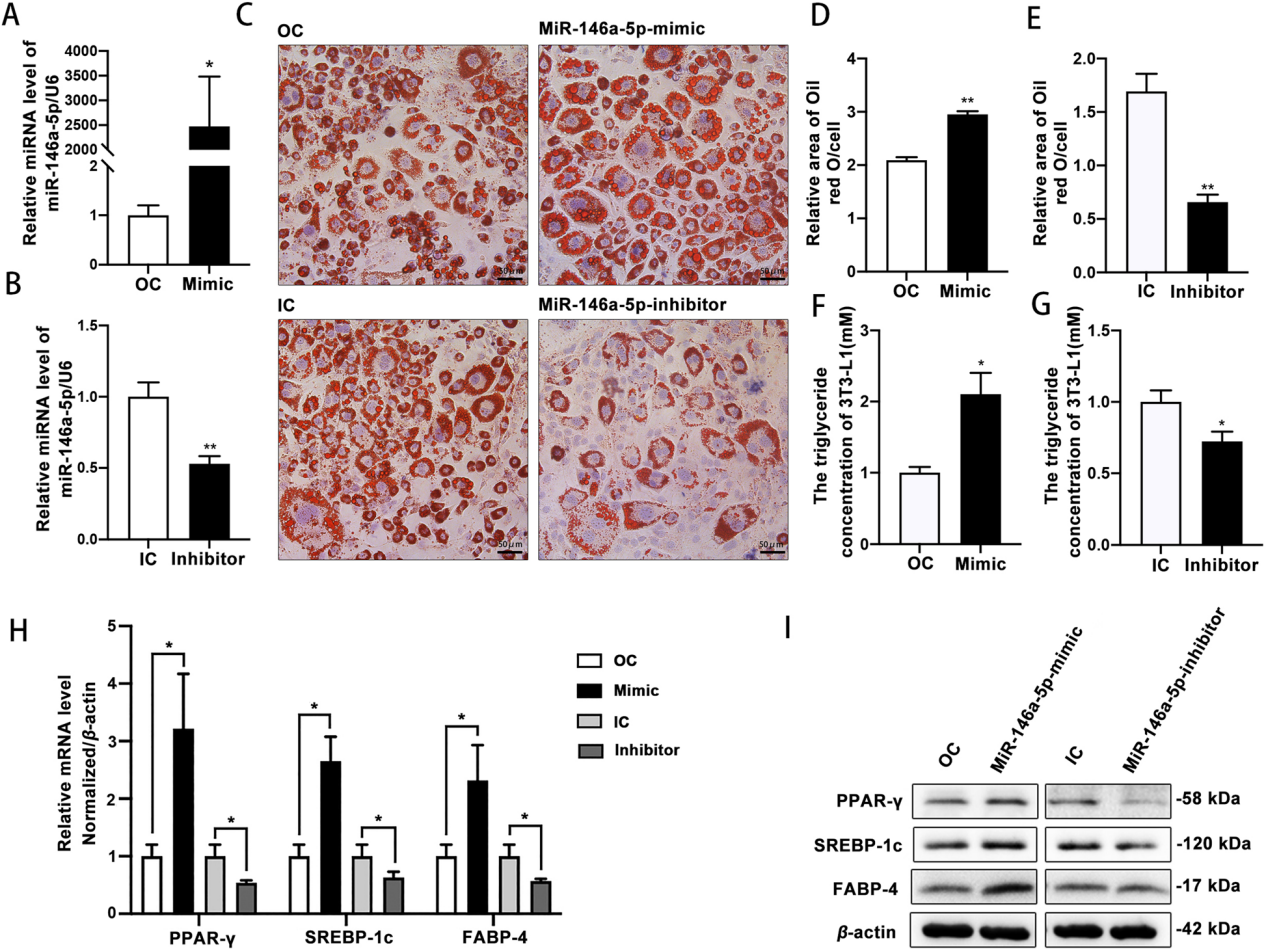
MiR-146a-5p promoted 3T3-L1 adipocyte differentiation. **A** MiR-146a-5p was overexpressed successfully in 3T3-L1 cells. **B** The expression of miR-146a-5p in the 3T3-L1 cells was inhibited successfully. **C**–**E** Oil Red O staining (200 ×) and relative areas of lipid droplets in miR-146a-5p–overexpressing and –inhibited 3T3-L1 cells after differentiation for 12 days. **F**, **G** Intercellular TG contents in miR-146a-5p–overexpressing and –inhibited 3T3-L1 cells after differentiation for 12 days. **H**, **I** Expression and protein levels of PPAR-γ, SREBP-1c, and FABP-4 in miR-146a-5p–overexpressing and –inhibited 3T3-L1 cells. Data in (**A**), (**B**), and (**D**–**H**) are means ± SDs (*n* = 3). ^*^P < 0.05, ^**^P < 0.01. OC, overexpression control; IC, inhibition control

MiR-146a-5p was overexpressed or down-regulated in 3T3-L1 cells successfully (Fig. 2A and B, Table 2), and the cells were induced for 12 days. The intracellular lipid droplet and TG contents were increased significantly in miR-146a-5p–overexpressing 3T3-L1 cells and decreased significantly in miR-146a-5p–inhibited 3T3-L1 cells (Fig. 2C, F, G). The relative areas of lipid droplets and the intracellular TG content were greater in miR-146a-5p–overexpressing 3T3-L1 cells than in miR-146a-5p–inhibited 3T3-L1 cells (Fig. 2D and E, Supplementary Fig. 1A and B). At the protein and mRNA levels, PPAR-γ, SREBP-1c, and FABP-4 expression was significantly increased in miR-146a-5p–overexpressing cells and decreased in miR-146a-5p–inhibited cells (Fig. 2H and I). These findings indicate that miR-146a-5p promoted 3T3-L1 cell differentiation.

**Table 2:** Nucleotide sequences of the miR-146a-5p mimic and inhibitor

Name	Sequences
miR-146a-5p mimic	UGAGAACUGAAUUCCAUGGGUU
miR-146a-5p inhibitor	AACCCATGGAATTCAGTTCTCA

### MiR-146a-5p targeted ErbB4 in 3T3-L1 cells

**Fig. 3:**
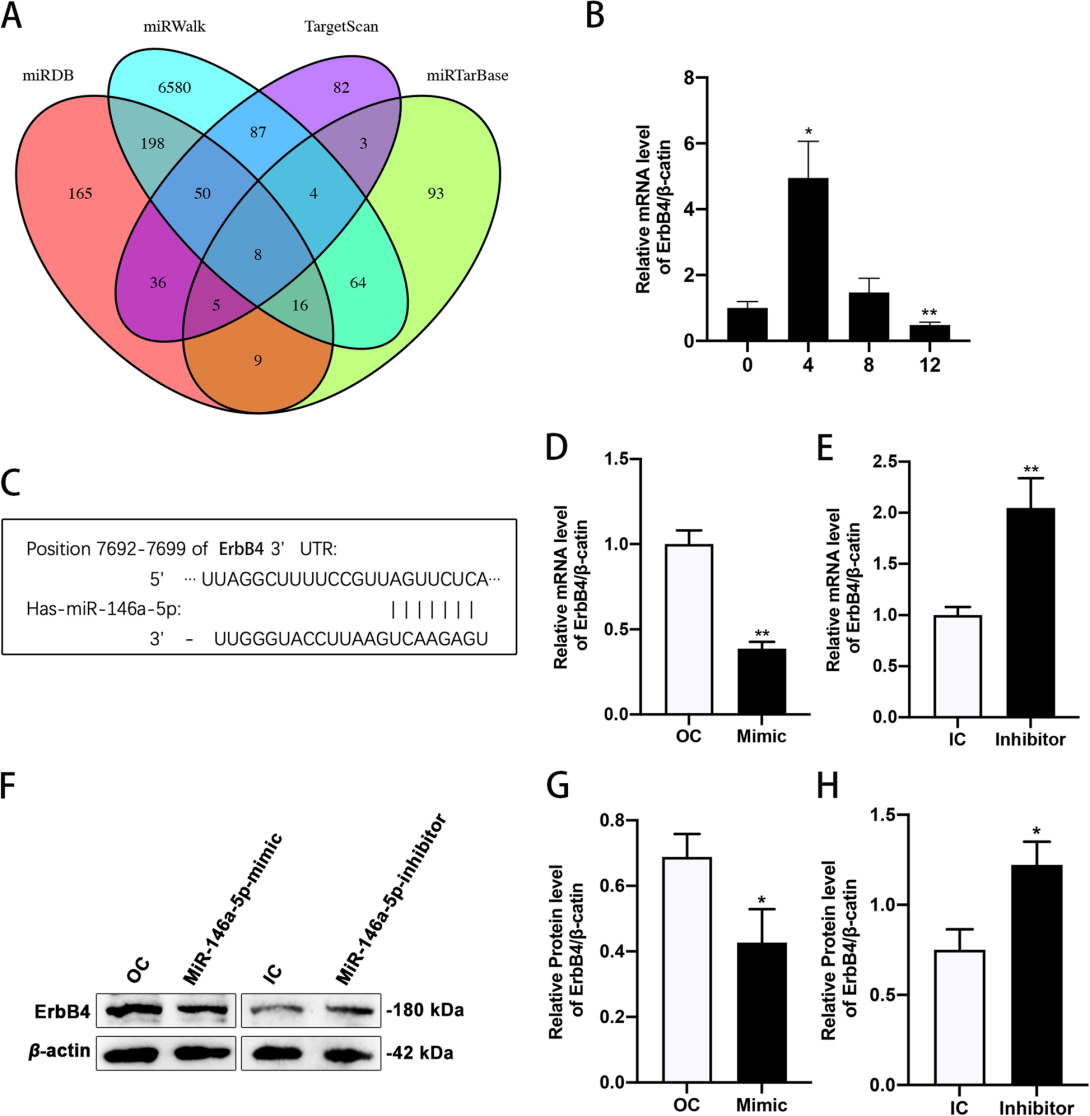
MiR-146a-5p targeted and regulated the expression of ErbB4 in 3T3-L1 cells. **A** Potential target genes of miR-146a-5p, predicted using the miRDB, miRTarBase, miRWalk and TargetScan online databases. **B** Expression of ErbB4 during 3T3-L1 cell differentiation. **C** Binding sites of miR-146a-5p and ErbB4, predicted using the TargetScan database. **D**, **E** Expression of ErbB4 in miR-146a-5p–overexpressing and –inhibited 3T3-L1 cells. **F**–**H** Relative protein levels of ErbB4 in miR-146a-5p–overexpressing and –inhibited 3T3-L1 cells. Data in (**B**), (**D**), (**E**), (**G**), and (**H**) are means ± SDs (*n* = 3). ^*^P < 0.05, ^**^P < 0.01. OC, overexpression control; IC, inhibition control

Eight genes including ErbB4 were identified as potential targets of miR-146a-5p (Fig. 3A). The ErbB4 level in 3T3-L1 cells was significantly elevated after 4 days of induction and markedly decreased after 12 days of induction, coinciding fully with the miR-146a-5p expression pattern (Fig. 3B). Predicted binding sites of miR-146a-5p and ErbB4 are shown in Fig. 3C. ErbB4 expression was significantly decreased in miR-146a-5p–overexpressing 3T3-L1 cells and markedly increased in miR-146a-5p–inhibited 3T3-L1 cells (Fig. 3D–H). These results suggest that miR-146a-5p interacts directly with ErbB4 and regulates its expression in 3T3-L1 cells.

### MiR-146a-5p promoted preadipocyte differentiation through the ERK1/2/PPAR-γ signaling pathway

**Fig. 4:**
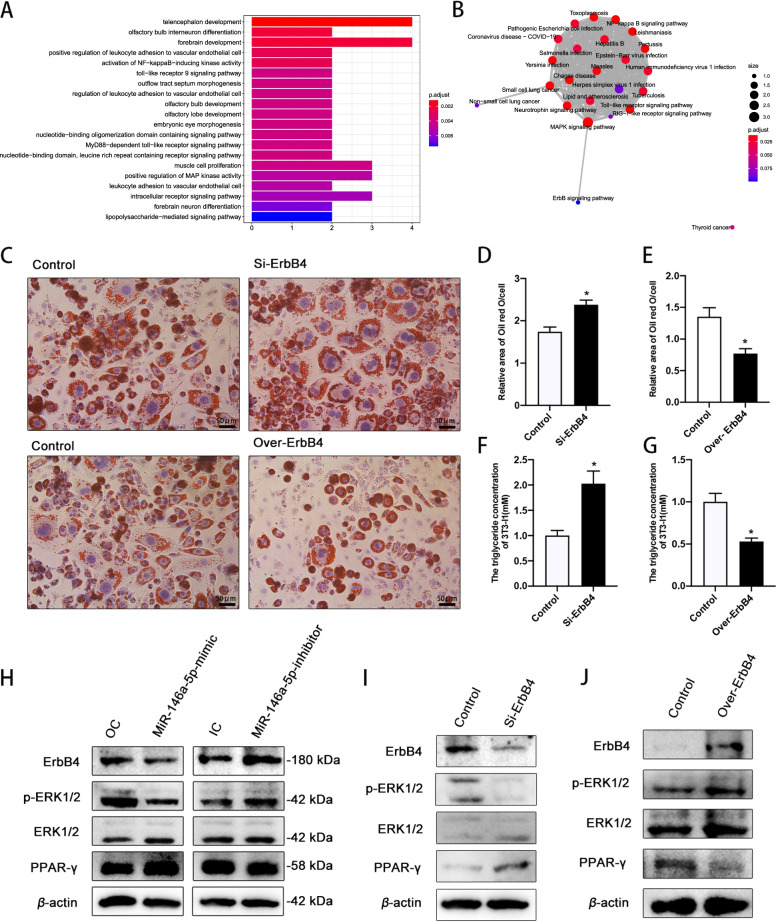
MiR-146a-5p promoted adipocyte differentiation through the ErbB4/ERK1/2/PPAR-*γ* signaling pathway. **A**, **B** GO (Biological Process) and KEGG enrichment maps of eight predicted target genes of miR-146a-5p. **C** Oil Red O staining of ErbB4-inhibited and -overexpressing 3T3-L1 cells after differentiation for 12 days (200×). **D**, **E** Relative areas of lipid droplets in ErbB4-inhibited and -overexpressing 3T3-L1 cells after differentiation for 12 days. **F**, **G** Intercellular TG contents of ErbB4-inhibited and -overexpressing 3T3-L1 cells after differentiation for 12 days. **H** Protein levels of ErbB4/ERK1/2/PPAR-γ signaling pathway components in miR-146a-5p–overexpressing and –inhibited 3T3-L1 cells. **I**, **J** Protein levels of ErbB4/ERK1/2/PPAR-γ signaling pathway components in ErbB4-inhibited and -overexpressing 3T3-L1 cells. Data in **D**–**G** are means ± SDs (*n* = 3). ^*^P < 0.05. OC, overexpression control; IC, inhibition control

**Fig. 5:**
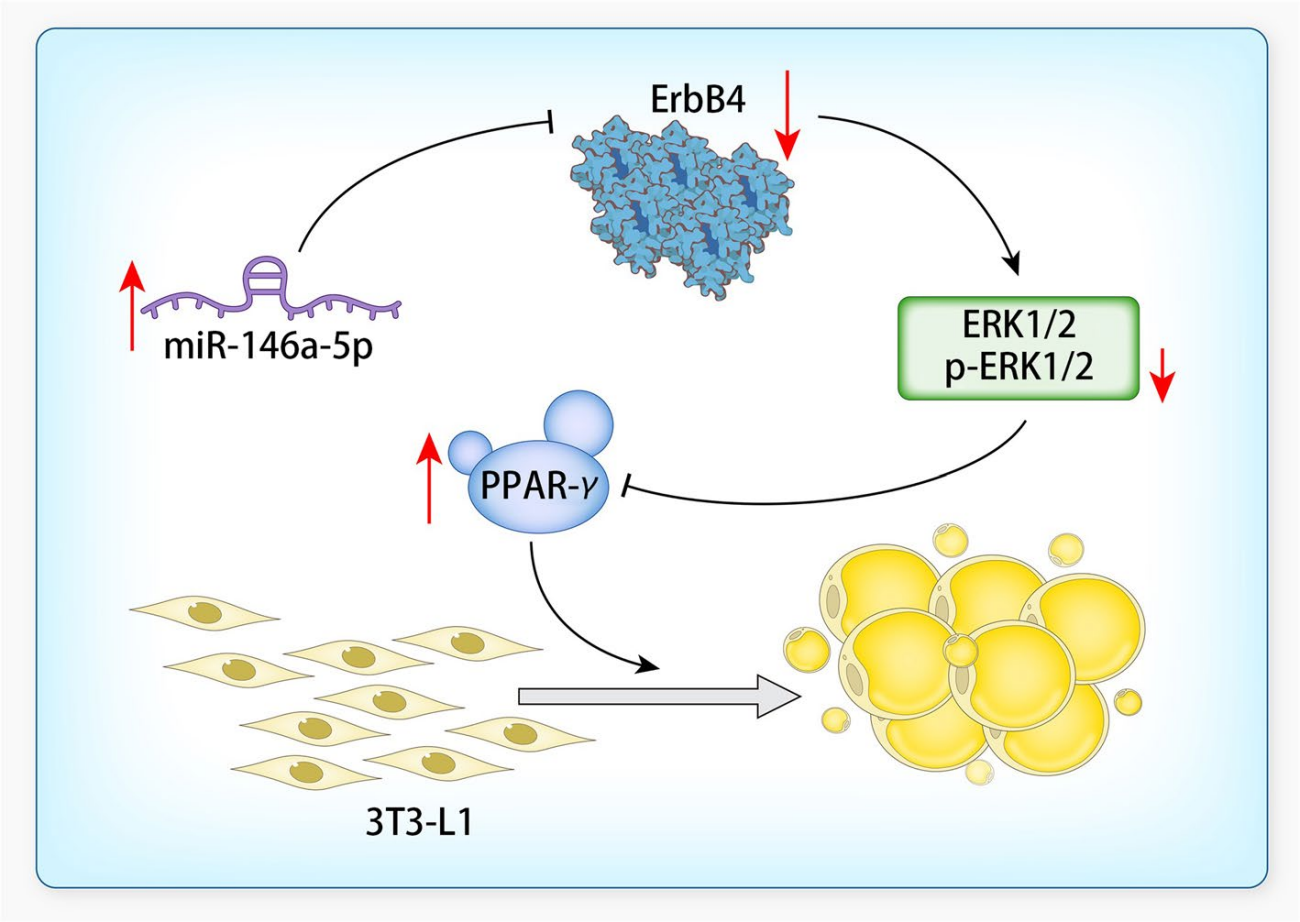
Proposed model of miR-146a-5p’s promotion of 3T3-L1 preadipocyte differentiation into mature adipocytes

The results of the GO and KEGG analyses suggest that the eight target genes were involved in the positive regulation of MAP kinase activity, and that ErbB4 interacts with the MAPK signaling pathway (Fig. 4A and B). As miR-146a-5p was found to regulate ErbB4 expression, it may promote preadipocyte differentiation through the ERK1/2/PPAR-γ signaling pathway. ErbB4 was inhibited or overexpressed in 3T3-L1 cells, followed by differentiation for 12 days. The inhibition of ErbB4 expression significantly increased the intracellular lipid droplet and TG contents, and ErbB4 overexpression markedly decreased these contents, in differentiated 3T3-L1 cells (Fig. 4C–G). In addition, the OD values of extracted lipid droplets were increased in ErbB4-inhibited 3T3-L1 cells, and decreased in ErbB4-overexpressing 3T3-L1 cells compared with the control (Supplementary Fig. 2A and B). In miR-146a-5p–overexpressing 3T3-L1 cells, ERK1/2 phosphorylation and ErbB4 expression were markedly decreased and PPAR-γ expression was significantly increased; the opposite was observed in miR-146a-5p–inhibited 3T3-L1 cells (Fig. 4H). ERK1/2 phosphorylation was decreased and PPAR-γ expression was increased in ErbB4-inhibited 3T3-L1 cells, with the opposite observed in ErbB4-overexpressing 3T3-L1 cells (Fig. 4I and K). These results indicate that miR-146a-5p promoted preadipocyte differentiation through the ERK1/2/PPAR-γ signaling pathway (Fig. 5).

### Upregulated miR-146a-5p expression correlated with adipogenesis in vivo

**Fig. 6:**
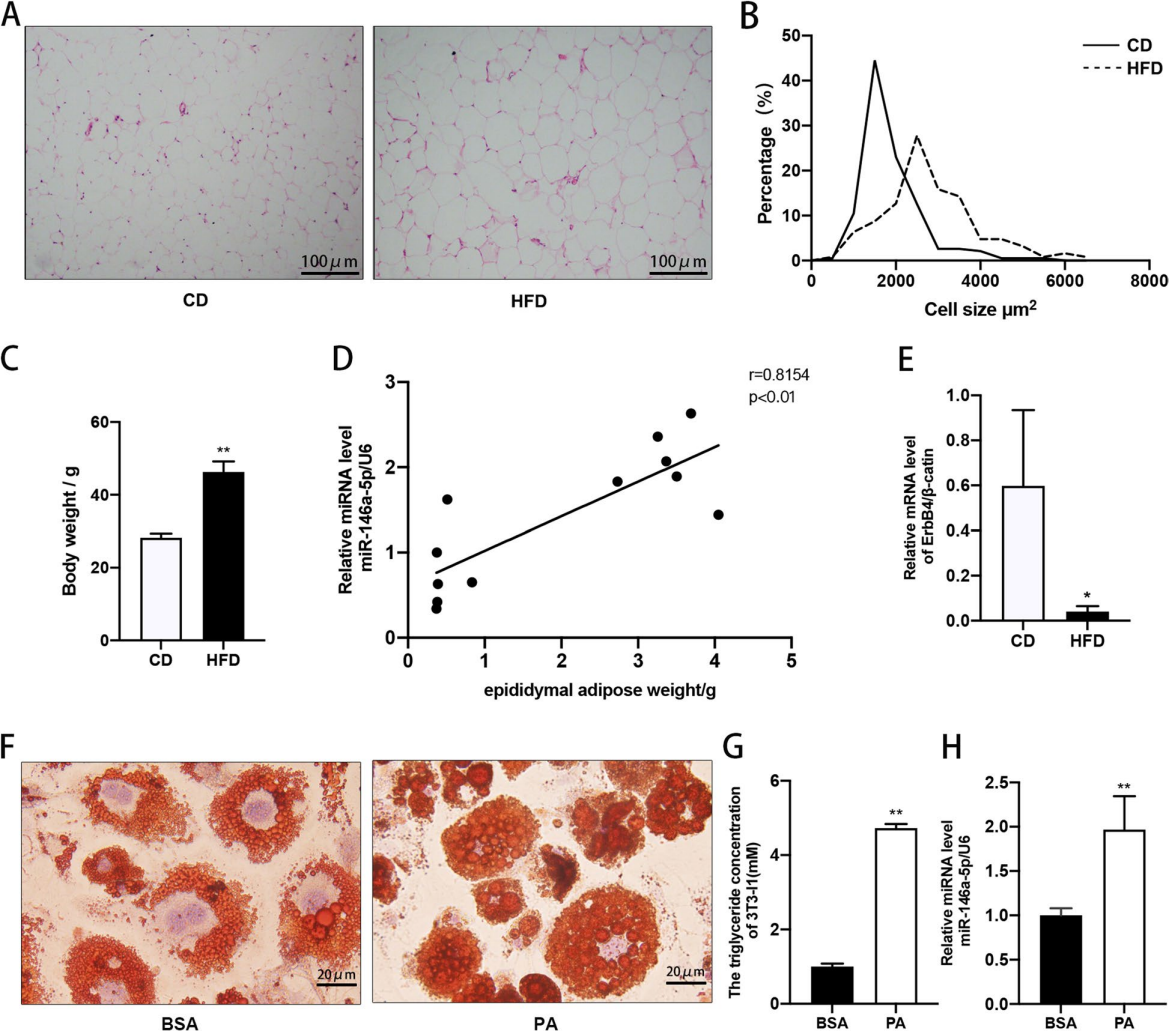
MiR-146a-5p expression correlated with adipogenesis in vivo. **A** HE staining of epididymal adipose tissues (100×). **B** Cell size profiles in epididymal fat mass. **C** Body weights of HFD-fed and control mice. **D** Correlation of miR-146a-5p expression with epididymal adipose tissue weight. **E** Relative expression of ErbB4 in epididymal adipose tissue from HFD-fed and control mice. **F** Oil Red O staining of the obese and control adipose cell models (400×). **G**, **H** Intracellular TG contents and relative miR-146a-5p expression in the obese and control adipose cell models. Data in (C–E), (G), and (H) are means ± SDs. ^*^P < 0.05, ^**^P < 0.01

HE staining revealed that the volumes of adipocytes from HFD-fed mice were larger than those of adipocytes from control mice (Fig. 6A and B). The HFD-fed mice had greater body weights than the control mice, and upregulated miR-146a-5p expression was associated with the epididymal adipose tissue weight (Fig. 6C and D). In addition, PPAR-γ, SREBP-1c, and FABP-4 expression was significantly greater in epididymal adipose tissue from HFD-fed mice than in that from CD-fed mice (Supplementary Fig. 3). ErbB4 expression was significantly lesser in the adipose tissue from HFD-fed mice (Fig. 6E). ORO staining of the obese mouse adipose cell model revealed significantly increased intracellular lipid droplet and TG contents relative to the control (Fig. 6F and G). In addition, miR-146a-5p expression was significantly increased in the model relative to the control (Fig. 6H). These results indicate that upregulated miR-146a-5p expression correlated with adipogenesis in vivo.

## Discussion

The effects of miR-146a-5p on preadipocyte differentiation, the main physiological process occurring during adipose tissue expansion, were investigated in this study. MiR-146a-5p expression increased during 3T3-L1 cell differentiation, promoting this process. This study also found that the promotion of adipocyte differentiation by miR-146a-5p likely occurs through the ERK1/2/PPAR-γ signaling pathway. In mice, miR-146a-5p upregulation correlated with adipogenesis. These findings suggest that miR-146a-5p plays an important role in preadipocyte differentiation.

MiRNAs affect adipocyte development and promote adipogenesis [41, 42]. Francisco et al. [18] investigated the miRNA expression profiles of human adipocytes during differentiation using global microarray analysis, and identified 70 miRNAs that were abnormally expressed in mature adipocytes compared with preadipocytes, indicating important crosstalk between miRNA expression and adipogenesis in vivo. MiR-214-3p has been found to regulate preadipocyte differentiation, during which key genes for lipogenesis are upregulated [43]. In this study, miR-146a-5p expression was markedly increased in differentiated 3T3-L1 cells. MiR-146a-5p overexpression promoted 3T3-L1 cell differentiation and enhanced mature adipocyte marker gene expression, whereas miR-146a-5p inhibition led to reduced cell differentiation and gene expression. These results are in accord with previous findings and indicate that miR-146a-5p promotes preadipocyte differentiation.

Many molecules and signaling pathways are known to be involved in preadipocyte differentiation. Yang et al. [44] observed marked miR-138 down-regulation during adipogenic differentiation, and reported that miR-138 negatively regulated the differentiation of human adipose tissue–derived mesenchymal stem cells (hAD-MSCs) by targeting EID-1. Chen et al. [35] reported that miR-125a-3p and miR-483-5p coordinated to promote adipogenesis through suppression of the RhoA/ROCK1/ERK1/2 signaling pathway in hAD-MSCs. In the present study, ErbB4 was determined to be the miR-146a-5p target gene, with an accompanying regulatory effect. Chen et al. [35] found that miR-125a-3p promoted adipogenesis by suppressing the phosphorylation of ERK1/2 and increasing the expression of PPAR-γ. KEGG enrichment analysis indicated that ErbB4 interacts with the ERK signaling pathway. In this study, miR-146a-5p overexpression reduced ERK1/2 phosphorylation and ErbB4 expression and increased PPAR-γ expression. Thus, miR-146a-5p appears to target ErbB4 to promote the differentiation of 3T3-L1 cells via the ERK1/2/PPAR-γ signaling pathway.

In contrast to these findings, Zhang et al. [45] found that miR-146a-5p expression first increased and then decreased during porcine intermuscular preadipocyte differentiation. A possible reason for this difference is that *in-vivo* preadipocyte differentiation is affected by adjacent tissues and multiple hormones. In this study, the effects of miR-146a-5p were further verified by the in vivo experiment, the results of which were in accordance with the in vitro results. GEO database analysis revealed greater expression of miR-146a-5p in human adipocytes than in preadipocytes (Supplementary Fig. 4A and B). Thus, multiple regulatory mechanisms may participate in preadipocyte differentiation in vivo.

### Comparisons with other studies and what does the current work add to the existing knowledge

Two findings from the current work are new: a) that miR-146a-5p promotes 3T3-L1 cell differentiation; b) by regulating the ERK1/2/PPAR-γ signaling pathway.

### Study strengths and limitations

This study has several strengths. It is the first to characterize the role of miR-146a-5p in preadipocyte differentiation, showing that miR-146a-5p upregulation correlated positively with 3T3-L1 cell differentiation and miR-146a-5p promoted 3T3-L1 cell differentiation via the ERK1/2/PPAR-γ signaling pathway. Several limitations of this study should be acknowledged. First, effects on the ERK1/2/PPAR-γ signaling pathway were not examined in epididymal adipose tissue from obese mice. Second, the interaction between miR-146a-5p and ErbB4 was not examined using a luciferase reporter gene assay. Further studies should be conducted to examine the role of miR-146a-5p in preadipocyte differentiation in vivo.

## Conclusions

This study illustrated the key role of miR-146a-5p in preadipocyte differentiation, which suggests that miR-146a-5p could serve as a biomarker in early screening for obesity and a therapeutic target for obesity and related diseases. Clinicians can predict the risk of obesity development by examining miR-146a-5p expression in vivo.

## Supplementary information

**Additional file 1.**

## Data Availability

All data generated or analyzed in this study are available from the corresponding author for the reasonable request.

## Abbreviations

BSA: Bovine serum albumin
DEX: Dexamethasone
DLK1: Delta like non-canonical notch ligand 1
DMEM: Dulbecco’s modified Eagle’s medium
ErbB4 (also named as EGFR4): Epidermal growth factor receptor-4
FABP-4: Fatty Acid Binding Protein 4
FBS: Fetal bovine serum
hAD-MSCs: Human adipose tissue-derived mesenchymal stem cells
HE: Hematoxylin–eosin
HFD: High-fat diet
IBMX: 3-Isobutyl-1-methylxanthine
MiRNAs: Micrornas
NAFLD: Non-alcoholic fatty liver disease
NCS: Newborn calf serum
ORO: Oil Red O
PA: Palmitic acid
PPAR-γ: Peroxisome Proliferator-Activated Receptor γ
P/S: Penicillin–streptomycin
qRT-PCR: Quantitative real-time polymerase chain reaction
ROS: Rosiglitazone
SD: Standard deviations
SDS: Sodium dodecyl sulfate
SREBP-1c: Sterol regulatory element binding protein 1c
TBST: Tris–HCl buffered saline containing 0.1% Tween -20
TG: Triglyceride
