# Supplementary material for: MiR-146a-5p, targeting ErbB4, promotes 3T3-L1 preadipocyte differentiation through the ERK1/2/PPAR-γ signaling pathway
Source: Lipids Health Dis. 2022 Jun 15;21:54. doi: 10.1186/s12944-022-01662-6 (PMC9202118; doi:10.1186/s12944-022-01662-6)
Supplement: Supplementary file 1 — Additional file 1. [file 12944_2022_1662_MOESM1_ESM.docx]

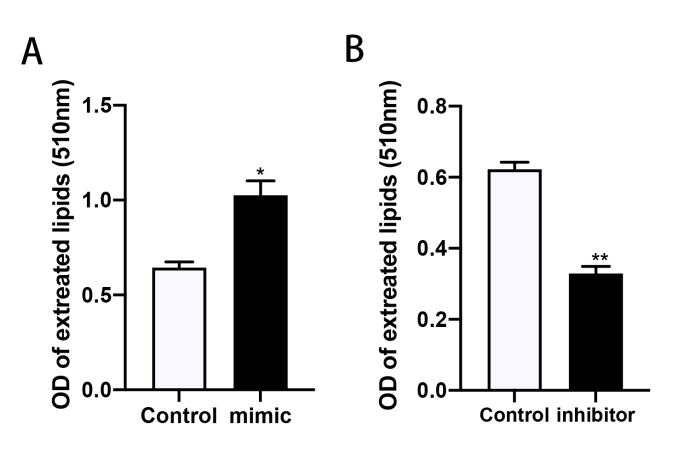
**Supplementary figures**


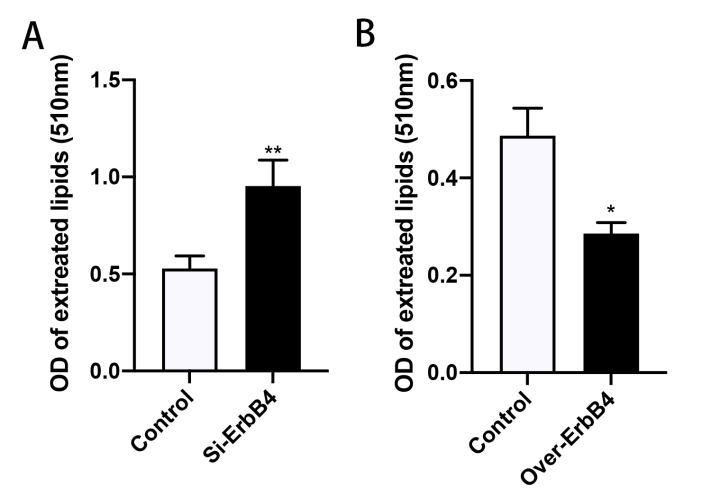
**Supplementary Figure 1.** OD values (mean ± SD) for lipids extracted from miR-146a-5p–overexpressing and –inhibited 3T3-L1 cells. ^*^*P* < 0.05, ^**^*P* < 0.01.


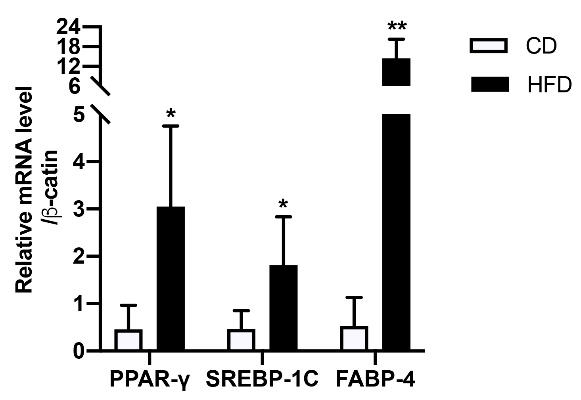
**Supplementary Figure 2.** OD values (mean ± SD) for lipids extracted from ErbB4-inhibited and -overexpressing 3T3-L1 cells. ^*^*P* < 0.05, ^**^*P* < 0.01.

**Supplementary Figure 3.** Relative expressions of PPAR-γ, SREBP-1c, and FABP-4 in epididymal adipose tissue of HFD-fed mice and CD-fed mice. ^*^*P* < 0.05, ^**^*P* < 0.01.


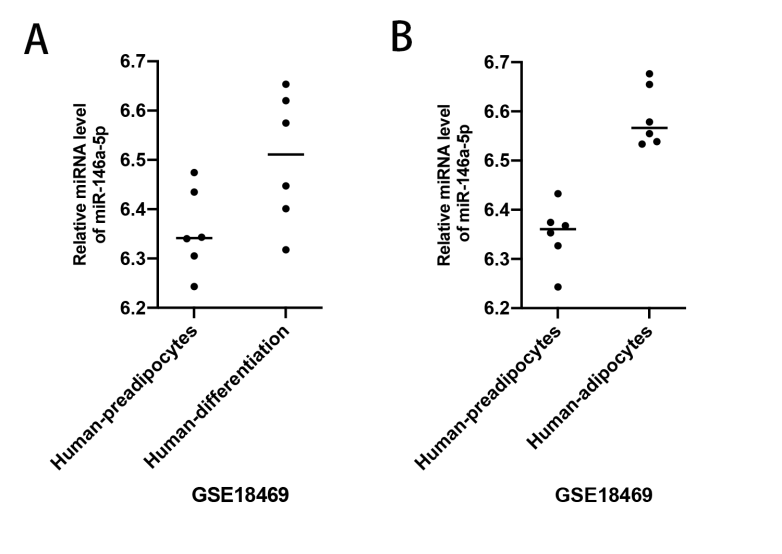
**Supplementary Figure 4.** Expression of miR-146a-5p in human preadipocytes and differentiated adipocytes after 7 (A) and 14 (B) days of differentiation.

**Supplementary methods**

**GEO database analysis**

MiRNAs have been found to be differentially expressed during adipogenesis in humans. Data on the expression of miR-146a-5p in healthy human subcutaneous adipose tissue on days 0, 7, and 14 of differentiation were acquired from a dataset with the accession number GSE18469 in the GEO database.
